# The Mitochondrial Ca^2+^ Uniporter MCU Is Essential for Glucose-Induced ATP Increases in Pancreatic β-Cells

**DOI:** 10.1371/journal.pone.0039722

**Published:** 2012-07-19

**Authors:** Andrei I. Tarasov, Francesca Semplici, Magalie A. Ravier, Elisa A. Bellomo, Timothy J. Pullen, Patrick Gilon, Israel Sekler, Rosario Rizzuto, Guy A. Rutter

**Affiliations:** 1 Section of Cell Biology, Division of Diabetes Endocrinology and Metabolism, Department of Medicine, Imperial College London, London, United Kingdom; 2 Institut de Génomique Fonctionnelle, INSERM U661, CNRS UMR5203, Université Montpellier I et II, Montpellier, France; 3 Pole of Endocrinology, Diabetes and Nutrition, Faculty of Medicine, Université Catholique de Louvain, Brussels, Belgium; 4 Department of Physiology, Faculty of Health Sciences, Ben Gurion University, Beer-Sheva, Israel; 5 Department of Biomedical Sciences, University of Padua, Padua, Italy; Université Joseph Fourier, France

## Abstract

Glucose induces insulin release from pancreatic β-cells by stimulating ATP synthesis, membrane depolarisation and Ca^2+^ influx. As well as activating ATP-consuming processes, cytosolic Ca^2+^ increases may also potentiate mitochondrial ATP synthesis. Until recently, the ability to study the role of mitochondrial Ca^2+^ transport in glucose-stimulated insulin secretion has been hindered by the absence of suitable approaches either to suppress Ca^2+^ uptake into these organelles, or to examine the impact on β-cell excitability. Here, we have combined patch-clamp electrophysiology with simultaneous real-time imaging of compartmentalised changes in Ca^2+^ and ATP/ADP ratio in single primary mouse β-cells, using recombinant targeted (*Pericam* or *Perceval*, respectively) as well as entrapped intracellular (Fura-Red), probes. Through shRNA-mediated silencing we show that the recently-identified mitochondrial Ca^2+^ uniporter, MCU, is required for depolarisation-induced mitochondrial Ca^2+^ increases, and for a sustained increase in cytosolic ATP/ADP ratio. By contrast, silencing of the mitochondrial Na^+^-Ca^2+^ exchanger NCLX affected the kinetics of glucose-induced changes in, but not steady state values of, cytosolic ATP/ADP. Exposure to gluco-lipotoxic conditions delayed both mitochondrial Ca^2+^ uptake and cytosolic ATP/ADP ratio increases without affecting the expression of either gene. Mitochondrial Ca^2+^ accumulation, mediated by MCU and modulated by NCLX, is thus required for normal glucose sensing by pancreatic β-cells, and becomes defective in conditions mimicking the diabetic milieu.

## Introduction

Glucose-induced insulin secretion from pancreatic β-cells is essential to ensure the normal control of blood glucose concentrations [1]. Defects in β-cell glucose sensitivity [2], [3] as well as a decrease in β-cell mass [4] are cardinal aspects of type 2 diabetes mellitus (T2D). A key event in glucose-induced insulin release is the stimulation of mitochondrial oxidative metabolism [5], [6]. Enhanced ATP synthesis [7] results in the closure of ATP-sensitive K^+^ (K_ATP_) channels [8], membrane depolarisation and Ca^2+^ influx via voltage-gated Ca^2+^ channels, which triggers insulin release [1], [9].

In most mammalian cells, mitochondrial oxidative metabolism is thought to be stimulated by Ca^2+^ [10], [11] through the activation of intramitochondrial dehydrogenases [12]. This stimulates the supply of reducing equivalents to the respiratory chain [13], and hence ATP synthesis [14]. The above process is thought also to be important in pancreatic β-cells [15] and recent analyses using a mitochondrial Ca^2+^ buffer [14] have suggested that mitochondrial Ca^2+^ accumulation is important for sustained insulin secretion.

The interplay between cytosolic Ca^2+^, mitochondrial Ca^2+^ and ATP synthesis has nonetheless remained enigmatic in the β-cell. In particular, Ca^2+^ entry into the cytosol, triggered by elevated ATP, is expected to enhance ATP hydrolysis, for example by activating granule exocytosis [16] and Ca^2+^ ATPases which pump the cation out of the cytosol [17]. The Ca^2+^-induced drop in ATP is then predicted to open K_ATP_ channels, thereby arresting Ca^2+^ influx [18]. In addition, Ca^2+^ has been suggested to induce repolarisation of the plasma membrane by opening Ca^2+^-activated K^+^ channels [19] or depolarising the mitochondrial inner membrane, which decreases the driving force for ATP synthesis by the F_1_F_o_ ATPase [20].

Until very recently, the molecular entities responsible for catalysing mitochondrial Ca^2+^ uptake have remained unclear in any mammalian cell type. However, two reports in 2011 identified a Ca^2+^-selective mitochondrial uniporter, MCU, encoded by the *Ccdc109a* gene [21], [22], in a complex with a Ca^2+^ sensing subunit MICU1 [23], as the likely Ca^2+^ transporting entity. Conversely, mitochondrial Ca^2+^ efflux was proposed to be mediated by the Na^+^-Ca^2+^ exchanger NCLX [24]. Whether these transporters catalyse mitochondrial Ca^2+^ transport in the β-cell, and may thus modulate insulin secretion, is currently unknown.

In the present study, we have sought to explore (a) the molecular mechanisms responsible for Ca^2+^ transfer across the mitochondrial membrane in β-cells and (b) the impact of these changes on cytosolic ATP dynamics and electrical excitability. To these ends, we have deployed a recently-developed, molecularly-addressed GFP-based recombinant probe for mitochondrial Ca^2+^ ([Ca^2+^]_mit_), 2mt8RP [25], alongside a trappable cytosolic Ca^2+^ probe (Fura Red) allowing us to image [Ca^2+^]_cyt_ simultaneously with [Ca^2+^]_mit_ in individual primary mouse β-cells. These measurements have been combined with perforated patch electrophysiology to allow plasma membrane potential (V_m_) to be recorded or controlled without perturbing cellular composition or metabolism [26]. Critically, this approach permits the ready and rapid control of [Ca^2+^]_cyt_ via voltage-gated Ca^2+^ channels [27] and thus an analysis of the interplay between [Ca^2+^]_cyt_ and [Ca^2+^]_mit_ in real time. In parallel, the novel ATP sensor *Perceval* [28], based on the bacterial regulatory protein, GlnK1, has been used to monitor the cytosolic ATP/ADP ratio ([ATP/ADP]_cyt_). These combined approaches have allowed us to characterise the roles of MCU and NCLX as regulators of mitochondrial ATP synthesis in the β-cell.

## Results

### Glucose induces a monophasic increase in cytosolic Ca^2+^ but a biphasic increase in cytosolic ATP/ADP ratio

We sought first to determine whether increases in [Ca^2+^]_cyt_ and/or [Ca^2+^]_mit_ might influence glucose-induced increases in [ATP/ADP]_cyt_. The latter parameter was therefore imaged in single mouse β-cells expressing the GFP-based probe *Perceval* [28], which was chiefly localised to the cytosol as expected (Suppl. Fig. S1A). Changes measured with this probe were shown to be unrelated to small alterations in cytosolic pH, and thus largely to reflect [ATP/ADP]_cyt_ (Suppl. Fig. S2A). [Ca^2+^]_cyt_ was imaged simultaneously in the same cell using the trappable cytosolic/nuclear probe Fura-Red (Suppl. Fig. S1A) whilst V_m_ was monitored using patch-clamp in current-clamp mode [3].

**Figure 1:**
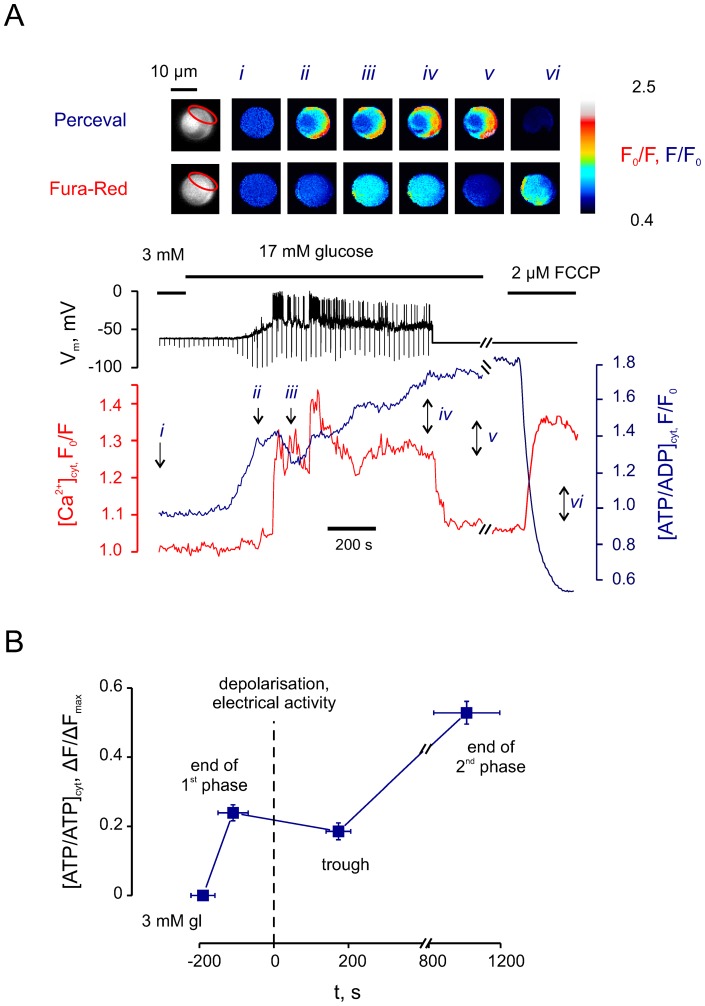
Glucose induces a biphasic increase in cytosolic ATP/ADP ratio. ***A***: The effects of high (17mM) glucose on [ATP/ADP]_cyt_ (reported with *Perceval*), [Ca^2+^]_cyt_ (Fura-Red) and V_m_ were measured in a single β-cell (representative of n = 30 cells). The voltage down-strokes were elicited by 10 ms 10 pA current injections applied every 20 s to monitor the input resistance which increased upon the elevation of [ATP/ADP]_cyt_. ***Inset***: Pseudo-colour images of the patched cell cluster presenting pixel-to-pixel ratios at the time points indicated by arrows (*i–vi*). ROI is indicated with red oval. Note that a cell expressing high levels of *Perceval* (just below the ROI) was deliberately excluded from analysis. ***B***: Characteristic times and amplitudes of glucose-induced [ATP/ADP]_cyt_ increase in β-cells (Fig. 1A; n = 30). The data were normalised to the width of the range of [ATP/ADP]_cyt_ change (ΔF_max_), measured as the difference in *Perceval* fluorescence between the peak point at 17 mM glucose and the point corresponding to application of 2 µM FCCP. Depolarisation and onset of electrical activity was taken as zero of the time axis. The change in [ATP/ADP]_cyt_ (ΔF/ΔF_max_) at each point is significant *vs* every other point (p<0.01, Wilcoxon's paired test).

β-Cells maintained at low (3 mM) glucose exhibited a resting V_m_ of −68±1 mV (*n* = 30, from 12 separate islet preparations; point *i* in Fig. 1A). An increase in glucose concentration to 17 mM led to a rapid elevation in [ATP/ADP]_cyt_ (Fig. 1A, point *ii*) and an increase in input resistance, followed by depolarisation of the plasma membrane and a [Ca^2+^]_cyt_ rise, as expected. This was closely followed by a drop in [ATP/ADP]_cyt_ (Fig. 1A, *iii*). The 33±4% drop (“trough” in Fig. 1B) was, however, transient and [ATP/ADP]_cyt_ quickly recovered and displayed a steady further increase (Fig. 1A, *iv*). The increase was not associated with any significant decrease in [Ca^2+^]_cyt_, and thus was not likely to reflect a lowering demand for Ca^2+^ extrusion or other ATP-consuming processes. Furthermore, setting V_m_ to −70mV via the patch pipette, thus closing voltage-gated Ca^2+^ channels, led to a prompt decrease in [Ca^2+^]_cyt_ (Fig. 1A, *v*). The application of the mitochondrial uncoupler carbonyl cyanide 4-(trifluoromethoxy)phenylhydrazone (FCCP) resulted in an abrupt decrease of [ATP/ADP]_cyt_, as expected (Fig. 1A, *vi*), and an elevation of [Ca^2+^]_cyt_, presumably due to a compromise in Ca^2+^ pumping across the plasma and ER membranes.

Combining data from multiple experiments (n = 30 single cells, Fig. 1B) we were able to observe that high glucose induced an [ATP/ADP]_cyt_ elevation in β-cells in two distinct phases (Fig. 1B). A rapid first phase preceded membrane depolarisation and electrical activity, whilst a slower second phase resulted in a larger increase of [ATP/ADP]_cyt_ (Fig. 1B). These changes contrasted with the essentially monophasic (albeit oscillatory) increases in [Ca^2+^]_cyt_ (Fig. 1A).

### Cytosolic Ca^2+^ influx is essential for the second phase of cytosolic ATP/ADP ratio increase

To dissect the dependence of the observed ATP increases on cytosolic Ca^2+^ increases prompted by depolarisation in response to glucose, we measured the changes in [ATP/ADP]_cyt_ in response to the sugar while keeping the cell hyperpolarised (V_m_ =  −70mV) using the patch pipette in voltage-clamp mode (as in point *v*, Fig. 1A). This prevented extracellular Ca^2+^ from entering the cytosol even at high extracellular glucose.

**Figure 2:**
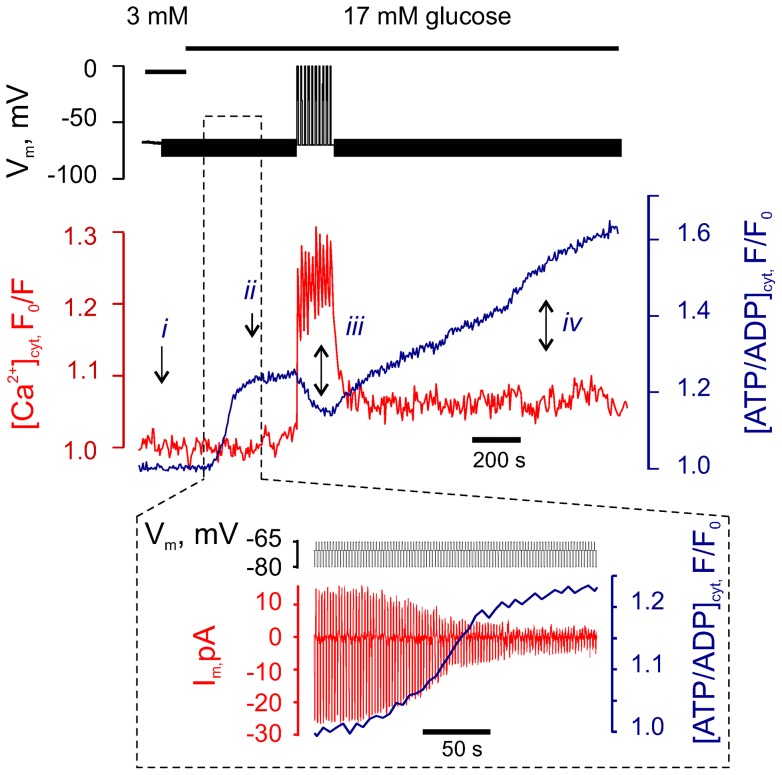
Ca^2+^ entry into the cytosol is essential for the biphasic increase of cytosolic ATP/ADP. The effect of high glucose on [ATP/ADP]_cyt_ and [Ca^2+^]_cyt_ was measured in a single β-cell voltage-clamped at −70 mV (representative of n = 12 cells). Small voltage steps (+5/−10 mV) were applied every second to measure the slow whole-cell current, I_m_. ***Inset***: dynamics of [ATP/ADP]_cyt_ and I_m_ during the indicated range corresponding to the first stage of ATP elevation.

An increase in glucose from 3 mM to 17 mM resulted in a rapid elevation of [ATP/ADP]_cyt_, followed by a saturation of the [ATP/ADP]_cyt_ level (*ii*, Fig. 2). Notably, in the absence of Ca^2+^ influx, neither a trough, nor an increase in [ATP/ADP]_cyt_ (see e.g. points *iii* and *iv* in Fig. 1A) were observed, suggesting that Ca^2+^ influx is involved in the latter changes. To test this possibility, we imposed forced changes in [Ca^2+^]_cyt_ with a train of 10 depolarisations (as given in Suppl. Fig. S2B) and then setting V_m_ back to −70mV (as indicated in the V_m_ trace in Fig. 2). The depolarisations triggered rapid and transient [Ca^2+^]_cyt_ elevation which, in turn, resulted in a transient drop in [ATP/ADP]_cyt_ (*iii*, Fig. 2) Remarkably, [ATP/ADP]_cyt_ started recovering while the depolarisation train was still being applied, at high [Ca^2+^]_cyt_, and this trend continued after V_m_ had been re-set to −70mV and [Ca^2+^]_cyt_ had decreased (*iv*, Fig. 2). These experiments indicate that the biphasic behaviour of [ATP/ADP]_cyt_ response to glucose is caused by the increase in [Ca^2+^]_cyt_ which results in a transient drop in [ATP/ADP]_cyt_ followed by its recovery. The two phases of the glucose-induced increase in [ATP/ADP]_cyt_ can therefore be classified as Ca^2+^-independent (the one that precedes) and Ca^2+^-dependent (the one that follows) Ca^2+^ entry.

We next sought to determine whether the apparent increases in cytosolic ATP/ADP ratio reported with *Perceval* were associated with the closure of ATP-sensitive K^+^ channels, as expected. This seemed an important question since fluctuations in “global” cytosolic ATP/ADP differ in some circumstances from those immediately beneath the plasma membrane, as recorded with a targeted luciferase-based probe [7]. The electrophysiological configuration used here allowed us to address this point as follows.

While keeping the cell hyperpolarised, at −70mV (Fig. 2), we applied small pulses between −65 and −80 mV, to monitor slow whole-cell current, I_m_. These pulses were too small to trigger any voltage-gated Ca^2+^ conductance and therefore had no effect on Ca^2+^ entry. The addition of 17 mM glucose decreased I_m_ during the Ca^2+^-independent phase of [ATP/ADP]_cyt_ increase (Fig. 2, inset), most likely due to the inhibition of K_ATP_ channels, the main providers of the β-cell conductance (G_m_) [29]. G_m_ thus was found to decrease from the initial value of 0.43±0.09 nS/pF to 0.09±0.02 nS/pF (n = 12) during the Ca^2+^-independent phase. A strong and significant correlation (Pearson's r =  −0.84±0.05, p<0.05, n = 12) between the elevation of [ATP/ADP]_cyt_ as recorded with *Perceval*, and the closure of K_ATP_ changes as measured above, (Suppl. Fig. S2C) indicated that the optical measurements with the GFP-based probe provided a useful guide to [ATP/ADP]_cyt_ changes in the physiologically-relevant domain beneath the plasma membrane. Interestingly, half-maximal inhibition of G_m_ coincided with the increase of [ATP/ADP]_cyt_ of 20±8% (n = 12, Suppl. Fig. S2C), while earlier data [29] suggest that half-maximal G_m_ is likely to be reached at around 28±4% of the [ATP/ADP]_cyt_ increase. Thus, the increase in [ATP/ADP]_cyt_ was reported with a 32±21 s delay after the drop in G_m_ measured using patch-clamp. This small delay may reflect the propagation of the glucose-induced ATP increase from the sub-membrane compartment to the bulk cytosol [7], [30].

### Glucose induces a sequential increase in [Ca^2+^]_cyt_ and [Ca^2+^]_mit_

**Figure 3:**
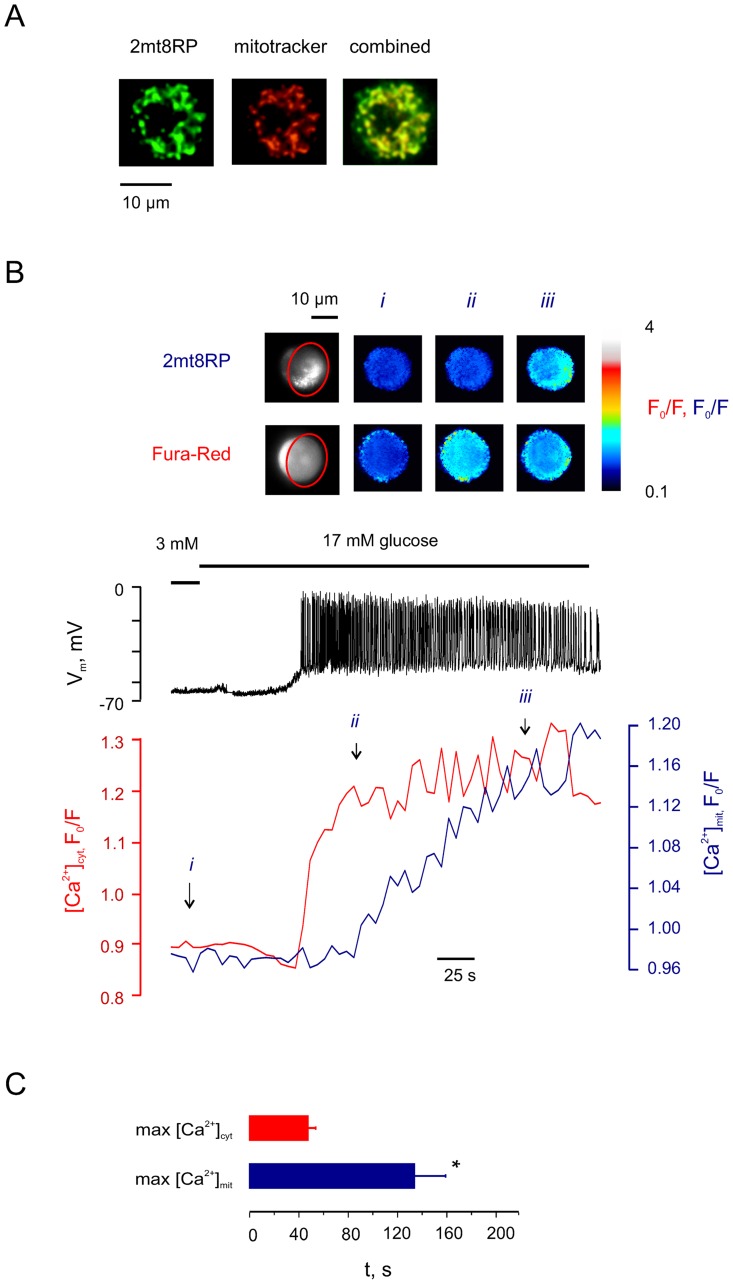
Mitochondrial [Ca^2+^] follows the increase in cytosolic [Ca^2+^] with a delay. ***A***: Colocalisation of 2mt8RP and Mitotracker Orange in a β-cell, 24 h post infection. ***B***: The effect of 17 mM glucose on V_m_, [Ca^2+^]_cyt_ (Fura-Red) and [Ca^2+^]_mit_ (2mt8RP) in a single pancreatic β-cell (representative of n = 10 cells). ***Inset***: Pseudo-colour images of the patched cell cluster presenting pixel-to-pixel ratios at the time points indicated by arrows (*i – iii*). ROI is indicated with red oval. ***C***: Mean times of maximal increase for [Ca^2+^]_cyt_ and [Ca^2+^]_mit_ in pancreatic β-cells, in response to 17 mM glucose (n = 10 cells). The times are calculated from the moment of the arrival of the first action potential. *Differences are statistically significant (p<0.01).

We next explored the possibility that the uptake of Ca^2+^ by mitochondria may be related to the second phase of [ATP/ADP]_cyt_ increase, as suggested by earlier experiments in β-cell populations [14]. To explore the temporal relationship between increases in [Ca^2+^]_cyt_ and [Ca^2+^]_mit_ in single β-cells after stimulation with glucose, we used a mitochondrial matrix-targeted fluorescent Ca^2+^ probe, 2mt8RP [25] (Fig. 3A; Supp. Fig. S1B). At 3 mM glucose, the plasma membrane was hyperpolarised as expected (V_m_ =  −68±1 mV, n = 22) and [Ca^2+^]_cyt_ and [Ca^2+^]_mit_ were stable (Fig. 3B, point *i*). Exposure to 17 mM glucose led to an increase in [Ca^2+^]_cyt_ (Fig. 3B, *ii*) which was followed later by an increase in [Ca^2+^]_mit_, presumably reflecting Ca^2+^ uniporter-mediated uptake (Fig. 3B, *iii*). [Ca^2+^]_cyt_ and [Ca^2+^]_mit_ reached their maximal amplitudes 47±6 s and 134±25 s, respectively, after the onset of glucose-induced electrical activity (Fig. 3C).

### MCU mediates mitochondrial Ca^2+^ increases and the second phase of glucose-induced [ATP/ADP]_cyt_ increases

**Figure 4:**
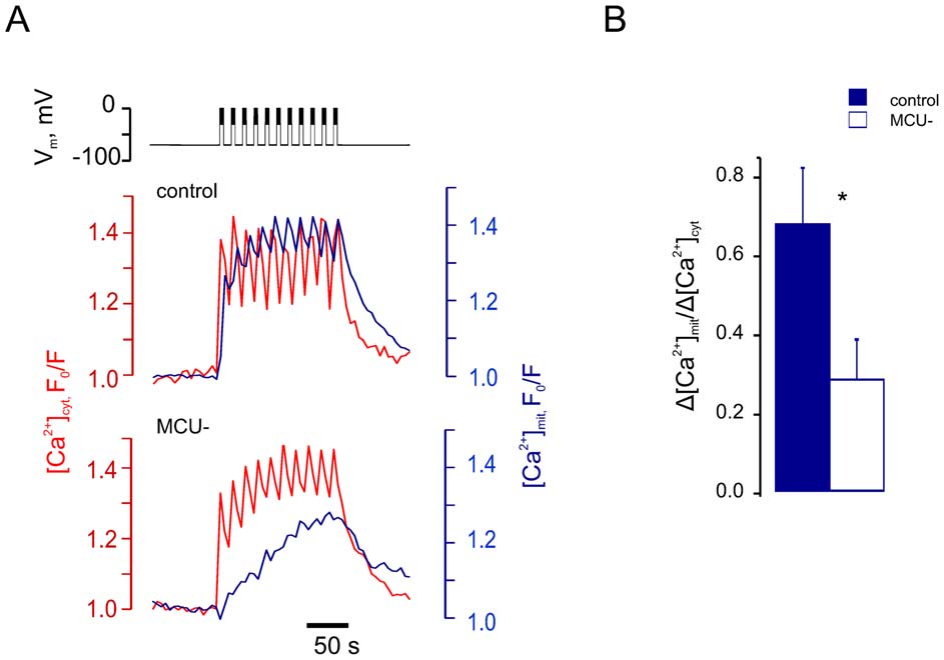
MCU silencing impairs mitochondrial Ca^2+^ increases. Pancreatic β-cells were infected with lentiviruses encoding nonsense (“control”) or anti-MCU (“MCU^−^”) shRNA for 72 h. ***A***: [Ca^2+^]_cyt_ (Fura-Red) and [Ca^2+^]_mit_ (2mt8RP) increases were measured in response to 10 depolarising bursts, applied at 4 min^−1^ by patch pipette (representative traces for n = 12, control, and n = 10, MCU^−^ cells). ***B***: Mean ratios of maximal increases in [Ca^2+^]_mit_ to the respective increases in [Ca^2+^]_cyt_ (Δ[Ca^2+^]_mit_/Δ[Ca^2+^]_cyt_) measured in control and MCU^−^ β-cells.

**Figure 5:**
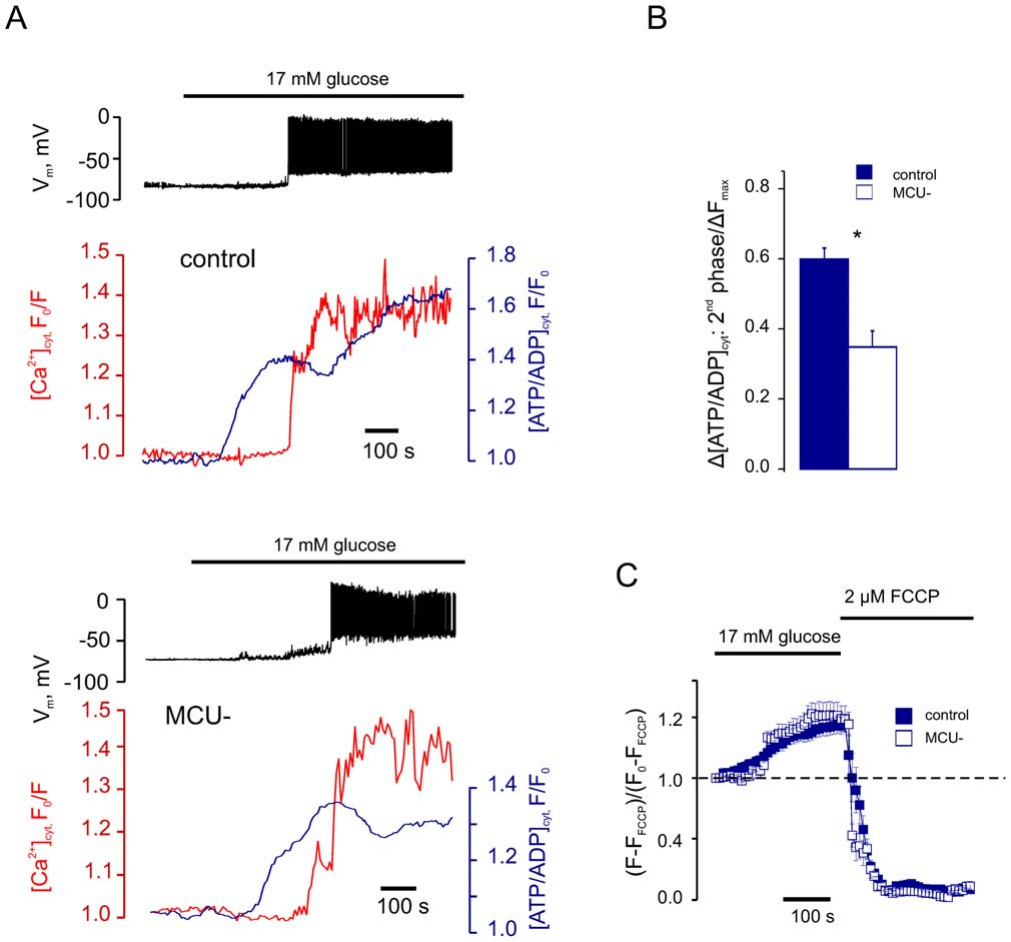
MCU silencing impairs the Ca^2+^-dependent phase of glucose-induced ATP increase. *A* : Glucose-induced changes in V_m_, [Ca^2+^]_cyt_ and [ATP/ADP]_cyt_ were measured in current clamp, using Fura-Red and *Perceval*, respectively (representative for n = 8, control, and n = 10, MCU^−^cells). ***B***: Mean magnitudes of the second phase of [ATP/ADP]_cyt_ increase measured in control and MCU^−^ β-cells. The data were normalised to the width of the range of [ATP/ADP]_cyt_ change (ΔF_max_), measured as the difference in *Perceval* fluorescence between the peak point at 17 mM glucose and the point corresponding to application of 2 µM FCCP. ***C***: Changes in ΔΨ_m_ measured as mitochondrial TMRE fluorescence, in response to the increase of glucose from 3 to 17 mM, in control and MCU^−^ β-cells. The data are expressed as (F-F_FCCP_)/(F_0_-F_FCCP_), where F_0_ and F_FCCP_ represent TMRE fluorescence intensity in 3 mM glucose and 2 µM FCCP, respectively. *Differences are statistically significant, p<0.01.

In experiments using an identical configuration to those above, the maximal rate of [ATP/ADP]_cyt_ decrease was observed 106±22 s after the first action potential (between points *ii* and *iii* in Fig. 1A). This observation, and those described for the time course of mitochondrial Ca^2+^ increases (Fig. 3B, C), are thus consistent with the possibility that mitochondrial Ca^2+^ accumulation (and hence an activation of oxidative metabolism) plays a role in the regulation of the [ATP/ADP]_cyt_ increase that follows an initial and small Ca^2+^-induced drop. To test this possibility directly we therefore reduced the expression of the recently-identified mitochondrial Ca^2+^ uniporter, MCU [21], [22], in β-cells by >80% (as assessed by qRT-PCR, not shown) using a lentivirally-delivered shRNA (Fig. 4). Silencing of MCU caused a substantial impairment of apparent Ca^2+^ entry into mitochondria, whilst the imposed cytosolic Ca^2+^ increases were unaffected (Fig. 4A, B). Importantly, this manipulation also resulted in an alteration of the glucose-induced [ATP/ADP]_cyt_ changes (Fig. 5A, B). Thus, MCU silencing had no effect on the first phase of the glucose-induced [ATP/ADP]_cyt_ increase, the rise of [Ca^2+^]_cyt_ or subsequent electrical spiking (Fig. 5A). However, the second (Ca^2+^-dependent) phase of the [ATP/ADP]_cyt_ increase, i.e. the [ATP/ADP]_cyt_ recovery, was significantly impaired in the β-cells where MCU expression was reduced (Fig. 5A, B).

To determine whether MCU knock-down might affect mitochondrial membrane potential (Ψ_m_) independently of a Ca^2+^ increase, we explored the glucose-induced changes in this parameter prior to [Ca^2+^]_cyt_ elevation using tetramethyl rhodamine, ethyl ester (TMRE). The resting Ψ_m_ (measured as −127±4 mV in control *vs* −133±5 mV in MCU^−^ cells) and the kinetics of the glucose-induced change (Fig. 5C) were not affected by the knock-down of MCU.

### NCLX modulates mitochondrial Ca^2+^ changes

**Figure 6:**
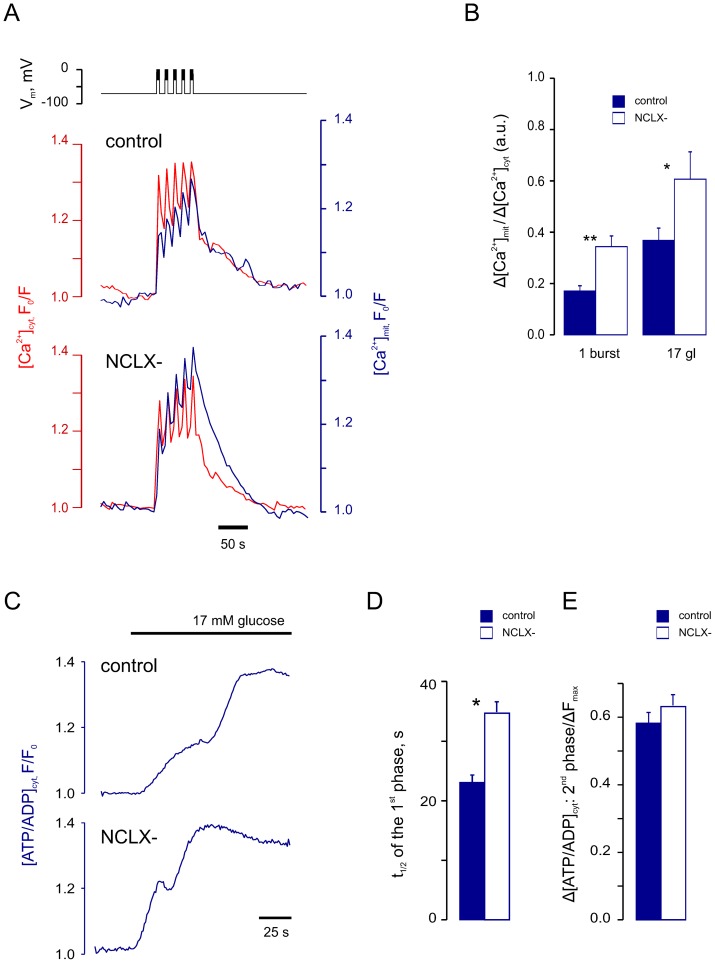
Effect of the NCLX silencing on [Ca^2+^]_cyt_ and [Ca^2+^]_mit_ dynamics. Pancreatic β-cells were infected with lentiviruses delivering nonsense shRNA (“control”) or shRNA against NCLX (“NCLX^-^“) for 36–48 h. ***A***: [Ca^2+^]_cyt_ and [Ca^2+^]_mit_ increases in response to 5 depolarising bursts applied at 4 min^−1^ were measured using Fura-Red and 2mt8RP, respectively. ***B***: Mean increases in [Ca^2+^]_mit_ induced by a single depolarising burst or by exposure to 17 mM glucose, related to the respective increases in [Ca^2+^]_cyt_ (Δ[Ca^2+^]mit/Δ[Ca^2+^]cyt). ***C***: Glucose-induced changes in [ATP/ADP]_cyt_ were measured using Perceval (representative for n = 9 control and n = 9 NCLX^−^ cells). ***D***: Times of half-maximal increase in [ATP/ADP]_cyt_ in response to 17 mM glucose, in control and NCLX^−^ cells. ***E***: Mean magnitudes of the second phase of [ATP/ADP]_cyt_ increase measured in control and NCLX^−^ β-cells. The data were normalised to the width of the range of [ATP/ADP]_cyt_ change (ΔF_max_), measured as the difference in *Perceval* fluorescence between the peak point at 17 mM glucose and the point corresponding to application of 2 µM FCCP. Differences vs respective NCLX^−^ data are significant with p<0.05 (*) or p<0.01 (**).

Pharmacological inhibition of mitochondrial Na^+^-Ca^2+^ exchange has been reported to elevate the basal ATP levels in INS-1 cells and primary rat islets [31]. However, the agent used (CGP37157) was likely to affect cellular Ca^2+^ homeostasis by targeting plasma membrane voltage-gated Ca^2+^ channels, as reported by Luciani *et* *al* [32]. NCLX was recently identified as an essential component of the mitochondrial Na^+^-Ca^2+^ exchanger [24], responsible for Ca^2+^ efflux from mitochondria, thereby providing an opportunity for a specific inhibition of Ca^2+^ efflux from mitochondria through RNA interference. In the present study, silencing of NCLX significantly potentiated depolarisation-induced increases in [Ca^2+^]_mit_ (Fig. 6A, B). NCLX silencing also slightly accelerated the onset of the first phase of the [ATP/ADP]_cyt_ response to glucose (Fig. 6C, D), but had no significant effect on the amplitude of the [ATP/ADP]_cyt_ changes (Fig. 6C, E).

### Chronic glucolipotoxicity inhibits mitochondrial Ca^2+^ increases and delays [ATP/ADP]_cyt_ recovery

Previous studies [33] have indicated that the structure and localisation of mitochondria are altered in β-cell dysfunction, including glucolipotoxicity, i.e. exposure to high levels of free fatty acids (FFA) and glucose. Importantly, glucose-induced ATP increases in the β-cell are impaired in this model of T2D [34]. We therefore sought to determine whether these changes were also associated with defective mitochondrial Ca^2+^ increases or altered expression of mitochondrial Ca^2+^ transporters.

**Figure 7:**
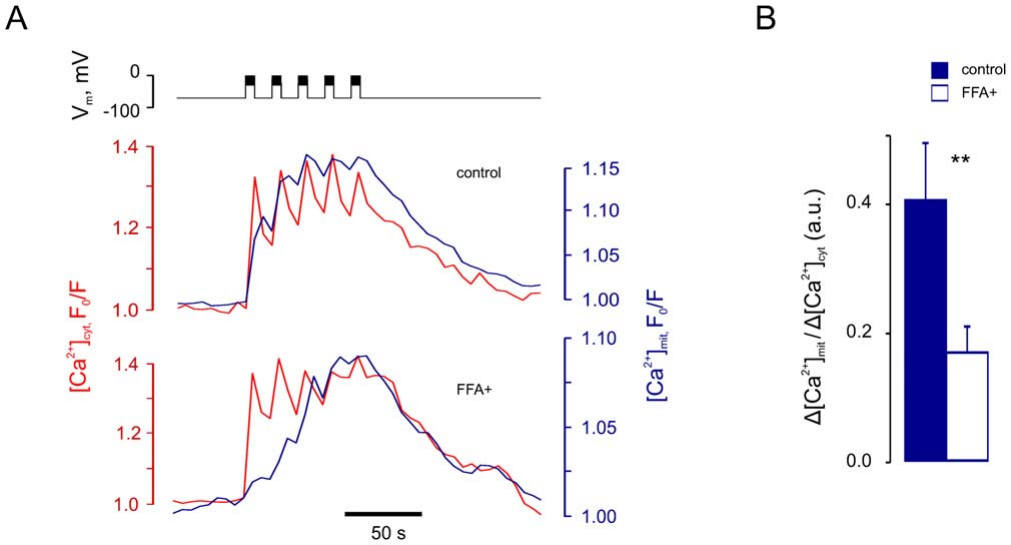
Chronic exposure to high-glucose and high-FFA medium impairs Ca^2+^ entry into mitochondria. β-Cells were pre-cultured in FFA-free medium containing 11 mM glucose (“control”) or medium containing 17 mM glucose and 0.5 mM palmitate (“FFA^+^”) for 48–72 h. ***A***: The cells were voltage-clamped at −70 mV and five depolarising bursts were applied at 4 min^−1^, as indicated in V_m_ trace (above). [Ca^2+^]_cyt_ and [Ca^2+^]_mit_ were monitored with Fura-Red and 2mt8RP, respectively. ***B***: Peak [Ca^2+^]_mit_ induced by a single burst related to the respective peak [Ca^2+^]_cyt_ (Δ[Ca^2+^]_mit_/Δ[Ca^2+^]_cyt_), measured in control (blue columns, n = 10) and FFA^+^ (white columns, n = 9) cells. *Differences are significant with p<0.05 (*) or p<0.01 (**).

**Figure 8:**
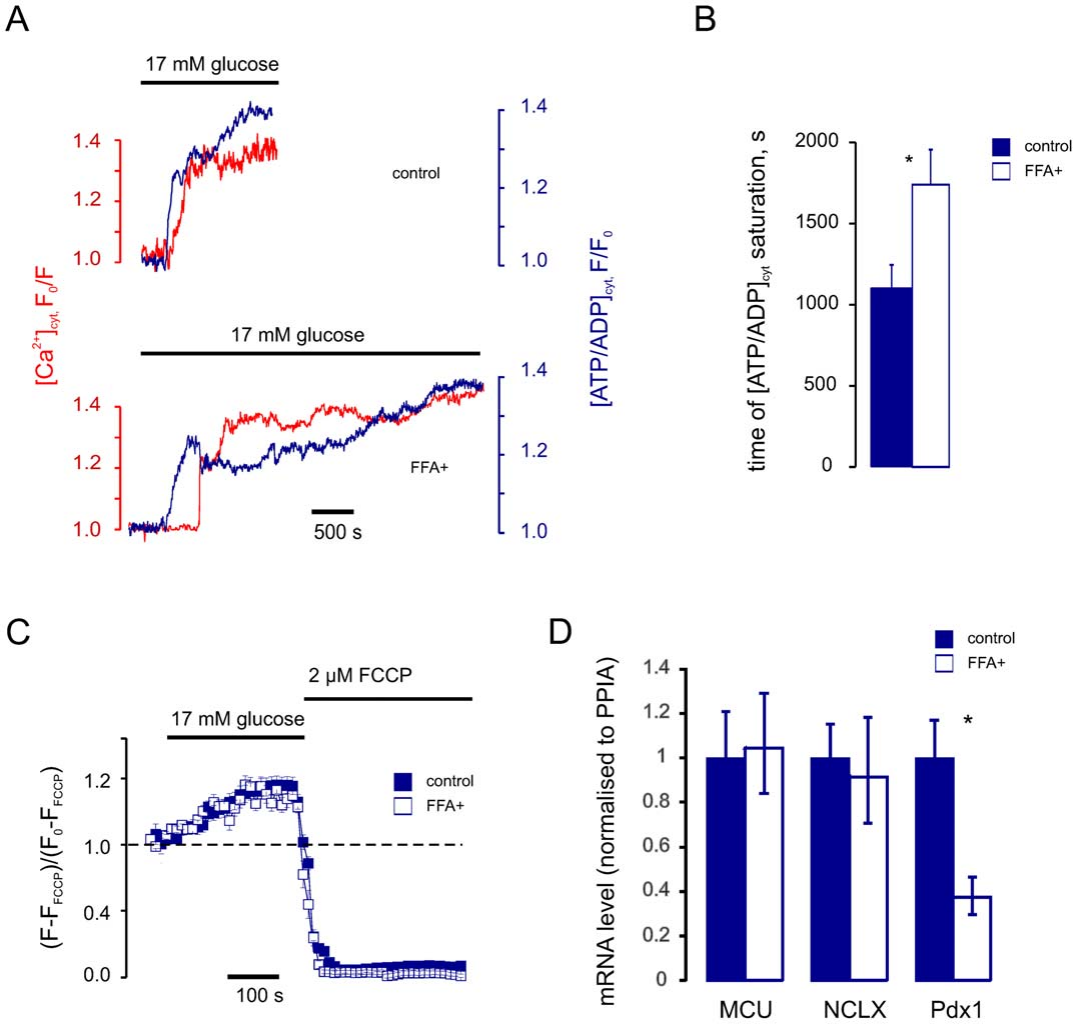
Chronic glucolipotoxicity slows down the second phase of glucose-induced ATP elevation. *A* : Glucose-induced changes in [ATP/ADP]_cyt_ and [Ca^2+^]_cyt_ were monitored in control (above) and FFA^+^ (below) cells using Perceval and Fura-Red. ***B***: Mean time of saturation of the second phase of [ATP/ADP]_cyt_ increase in control (blue columns, n = 16) and FFA^+^ (white columns, n = 13) cells. ***C***: Changes in ΔΨ_m_ measured as mitochondrial TMRE fluorescence, in response to the increase of glucose from 3 to 17 mM, in control and FFA^+^ β-cells. The data are expressed as (F-F_FCCP_)/(F_0_-F_FCCP_), where F_0_ and F_FCCP_ represent TMRE fluorescence intensity in 3 mM glucose and 2 µM FCCP, respectively. ***D***: Normalised MCU (*Ccdc109a*), NCLX (*Slc24a6*) and Pdx1 (*Pdx1*) mRNA expression levels for control and FFA^+^ cells. *Differences are significant (p<0.05).

To this end, we cultured primary mouse β-cells under glucolipotoxic conditions (“FFA^+^” cells) and studied the impact on the dynamics of [Ca^2+^]_cyt_ and [Ca^2+^]_mit_ in response to V_m_ manipulation. FFA^+^ cells displayed slower dynamics of [Ca^2+^]_mit_ increase (Fig. 7A, B). This resulted in a slower onset of the second phase of glucose-induced ATP increase (Fig. 8A, B) in FFA^+^ β-cells. This effect was not likely to be caused by changes in resting Ψ_m_ (−135±4 mV in control *vs* −137±4 mV in FFA^+^ cells) or the kinetics of the glucose-induced change in Ψ_m_ (Fig. 8C). We also failed to observe any significant change of either MCU or NCLX mRNA levels under these conditions (Fig. 8D). The expression of the transcription factor pancreatic duodenum *homeo*box-1 (Pdx1), in contrast, was significantly reduced by the chronic glucolipotoxicity, in line with earlier observations [35].

## Discussion

### Multiparametric analysis of glucose signalling in single primary β-cells

We dissect here the role of mitochondrial Ca^2+^ transport in the stimulation of single primary pancreatic β-cells with glucose using a combined imaging and electrophysiology approach. This has allowed us to monitor or manipulate up to four key parameters simultaneously in the same individual cell. Earlier studies in these cells combined the use of a microelectrode [36] or patch-clamp [37] with [Ca^2+^] measurements to report a close association of [Ca^2+^]_cyt_ and V_m_ signals during glucose-induced depolarisation. Furthermore, the control of V_m_ using perforated-patch was shown to be a very efficient means of rapid and precise control of [Ca^2+^]_cyt_ [19], [38]. The latter strategy provided a powerful tool here to explore the inter-relationships between Ca^2+^ changes in discrete compartments and with the control of ATP synthesis. Thus, a key technical advantage over earlier studies [14] has been the ability to resolve the exact sequence in which signalling events occurred within the same individual cell. Moreover, possible artefacts resulting from the progressive recruitment of cells within a population were also excluded.

These studies also represent the first use of the novel ATP/ADP probe *Perceval* [28] in an excitable cell, and provide significant advances over the previous use of less sensitive luciferase-based reporters [7], [39]. Although the affinity of *Perceval* for ATP is relatively high, competition with ADP lowers its sensitivity to a range appropriate for the β-cell cytosol (∼1 mM ATP at 3 mM glucose) [7], [29]. Importantly, pH changes appeared not to interfere with the probe (Suppl. Fig. S2A).

### MCU mediates mitochondrial Ca^2+^ uptake and enhanced ATP synthesis in pancreatic β-cells

We demonstrate here firstly that both cytosolic and mitochondrial Ca^2+^ increases are essential for the sustained (second) phase of [ATP/ADP]_cyt_ increase in response to high glucose. Interestingly, we show (Fig. 2) that a transient imposed increase in [Ca^2+^]_cyt_ is sufficient to lead to a progressive and sustained increase in [ATP/ADP]_cyt_. This finding is consistent with the possibility that mitochondrial uptake of Ca^2+^ in response to high glucose (which is slow compared to increases in cytosolic Ca^2+^; Fig. 3B, C) may then allow a sustained activation (i.e. “plasticity” or “memory”) of oxidative metabolism [39], [40].

Recent studies [21], [22], have provided convincing evidence for a role of MCU in mitochondrial transport in mammalian fibroblasts. However, no evidence currently exists demonstrating a role for this protein in this process in a more differentiated cell type. We report here firstly that MCU is critical for mitochondrial Ca^2+^ accumulation in pancreatic β-cells in response to depolarisation-induced Ca^2+^ increases. Likewise, we show that the Na^+^-Ca^2+^ exchanger NCLX [24] regulates [Ca^2+^]_mit_ increases and may thus be involved in regulating the responses to glucose, consistent with earlier findings using the pharmacological inhibitor CGP37157 [31]. Specifically, NCLX silencing affected the kinetics of the glucose-induced ATP/ADP changes but had no significant effect on the steady-state ATP/ADP level. Although the mechanisms underlying this unexpected observation are presently unclear, they may involve glucose-dependent changes in cytosolic [Na^+^] (unpublished observation of I.S.). Future studies are required to address this question and the role of NCLX in the β-cell.

**Figure 9:**
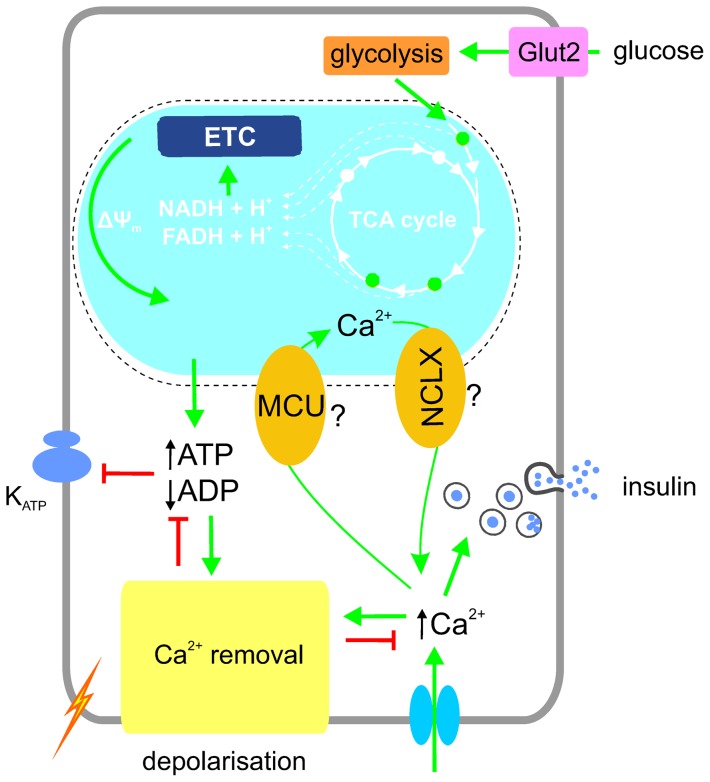
Proposed scheme of interplay between Ca^2+^, ATP and V_m_ in the β-cell. The oxidation of glucose that enters the β-cell hyperpolarises the mitochondrial membrane (ΔΨ_m_) thereby leading to the elevation of cytosolic ATP/ADP ratio, closing of K_ATP_ channels, depolarisation of the plasma membrane (V_m_) and Ca^2+^ entry. Elevated cytosolic [Ca^2+^] triggers a number of ATP-dependent processes including insulin secretion and Ca^2+^ removal into the ER and extracellular medium. By entering mitochondria via MCU, Ca^2+^ potentiates oxidative metabolism to counter-balance ATP expenditure. Ca^2+^ exits mitochondria via NCLX.

Overall, our data support a two-phase model (Fig. 9), in which an initial increase in cytosolic [ATP/ADP] (first phase) occurs independently of any increase in cytosolic (or mitochondrial) Ca^2+^ concentration. In the second phase, the elevation of cytosolic Ca^2+^ concentration leads to a gradual increase in mitochondrial Ca^2+^ (Fig. 3B). This, in turn, is likely to activate intramitochondrial dehydrogenases [10] (and perhaps other mitochondrial enzymes) [41], stimulating respiratory chain activity and hence mitochondrial ATP production. In line with this view, the initial rapid glucose-induced increase in [ATP/ADP]_cyt_ (first phase) was not affected by the MCU silencing whereas the second phase of [ATP/ADP]_cyt_ increase was essentially eliminated.

A recent study [14] also described biphasic increases in cytosolic ATP/ADP in β-cell populations in response to glucose, and indicated that mitochondrial Ca^2+^ accumulation may be essential for increases in cytosolic ATP/ADP in response to the sugar. However, this earlier study relied on the over-expression in the mitochondrial matrix of a high affinity (and high capacity) calcium-binding protein, S100G. Whether the presence of this protein within the mitochondrial matrix may interfere with normal mitochondrial function (for example by leading to a decrease in mitochondrial pH as a result of Ca^2+^ binding) is unclear.

### A role for MCU in the regulation of β-cell excitability and insulin secretion?

Mitochondrial Ca^2+^ accumulation, catalysed by MCU, is revealed here to be essential for the second phase of glucose-induced ATP synthesis by glucose. What may be the consequences for electrical activity and insulin secretion? Increases in ATP are believed to be involved in both “K_ATP_-dependent” and “K_ATP_-independent” regulation of exocytosis by glucose [16], [42]. Importantly, we obtained no evidence for a role for mitochondrial Ca^2+^ accumulation in the regulation of plasma membrane electrical activity (Fig. 5) suggesting that an involvement of mitochondrial Ca^2+^ in the regulation of insulin secretion, as implied by earlier studies [14], is likely to involve the latter (K_ATP_-independent) action on secretory granule movement or fusion, perhaps powered by ATP increases [43]. Further studies, using larger cell populations, will be necessary to explore the impact of MCU on phasic insulin secretion.

### A role for mitochondrial Ca^2+^ transport in β-cell glucolipotoxicity?

We show here that glucolipotoxic conditions impair Ca^2+^ transport into mitochondria (Fig. 7) and the second phase of glucose-induced ATP/ADP increases (Fig. 8). The expression of both MCU and NCLX was unaltered under these conditions (Fig. 8D), in line with previous studies in models of diet-induced β-cell dysfunction in rodents [44]. It is therefore likely that changes in the intracellular distribution of mitochondria induced by the diabetic milieu [33] are involved in this impairment in mitochondrial Ca^2+^ transport. These changes in mitochondrial architecture, and hence localisation at sites of Ca^2+^ entry into the cytosol [45], may consequently interfere with mitochondrial Ca^2+^ transport and ATP production.

### Conclusions

We show here that mitochondrial Ca^2+^ uptake in the excitable β-cell is mediated by MCU and modulated by NCLX. Changes in Ca^2+^ in the mitochondrial matrix are shown to be critical for increases in cytosolic ATP/ADP ratio, and may thus be required for glucose-stimulated insulin secretion [14]. Manipulation of MCU activity, in particular, may thus provide potential strategies to improve defective insulin secretion in some forms of diabetes.

## Materials and Methods

### Islet isolation and culture

Female CD1 mice were sacrificed by cervical dislocation as approved by the United Kingdom Home Office (HO) Animal Scientific Procedures Act, 1986 and designated as “Schedule 1” procedure. Animals were maintained under HO Licence PPL 70/7349 (Holder Dr I Leclerc), which received local ethical committee approval, and all participants received approved local training at Imperial College. Pancreatic islets were isolated by collagenase digestion [46], pre-cultured for 5 h in RMPI-1640 medium, containing 11 mM glucose, 10% FCS, 100 U penicillin, 100 μg streptomycin, at 37°C, 5%CO_2_, infected with an appropriate adenovirus encoding cDNA for the required probe, split into single β-cells and plated on glass coverslips. The cells were then cultured for >24 h in absolute humidity for 2–4 days and assayed as described below. Glass-attached single cells or 2-3-cell clusters displayed an infection efficiency of ∼90%. β-Cells were identified morphologically and according to their electrophysiological characteristics (membrane capacitance, V_m_, K_ATP_ current, lack of Na^+^ current, response to glucose).

Chronic glucolipotoxicity was modelled by culturing the cells in medium containing 0.5 mM Na^+^-palmitate and 17 mM glucose for 72 h. Palmitate was prepared as a 150 mM stock in ethanol; the working solution also contained 0.67% fatty-acid free BSA (Sigma). Control medium contained, respectively, 0.67% FFA-free BSA and 0.17% ethanol.

MCU was silenced in primary β-cells by 24h incubation with shRNA-bearing lentiviral particles (sc-142052-V, Santa-Cruz Biotechnology), at 1×10^6^ infectious units/ml. Cells infected with the GFP^+^ control particles (sc-108084) at the same titre displayed a multiplicity of infection of two, 36 hours after infection. Particles delivering non-target shRNA (sc-108080) were used as a negative control.

### Molecular biology and generation of adenoviruses

cDNA encoding Perceval [28] was excised from pGW1CMV-Perceval plasmid (kindly provided by Prof Gary Yellen, Yale University) by restriction first with *Eco*RI, then extension using T4 DNA-polymerase and finally by restriction with *Hin*dIII to liberate the insert. The *Hin*dIII/blunt insert was cloned into pShuttleCMV previously digested with *Eco*RV and *Hin*dIII.

cDNA encoding 2mt8-ratiometric pericam (2mt8RP) was kindly provided by Prof Tullio Pozzan (University of Padua). “Mt8” refers to the first 36 amino acids of subunit VIII of human cytochrome *c* oxidase (COX) while the targeting efficiency was improved by using two tandem repeats of the addressing sequence [25]. Adenoviral particles were produced as in [47].

### Gene expression measurement by qRT-PCR

RNA was purified from islet samples using Trizol. RNA was quantified by Nanodrop spectrophotometer then reverse transcribed using a High Capacity cDNA Reverse Transcription kit (Applied Biosystems). mRNA abundance was quantified by qPCR using Sybr Green PCR Master Mix (Applied Biosystems) on a 7500 Fast Real-time PCR machine. Expression of each gene was normalised to cyclophilin A (*Ppia*), and FFA treatment effect as fold change with 95% confidence intervals was calculated using the ΔΔC_T_ method on 7500 Software (Applied Biosystems, v2.0.5).

### Single cell epifluorescence imaging

Simultaneous imaging of [Ca^2+^] in mitochondria and the cytosol was performed using the mitochondrial pericam 2mt8RP, and Fura-Red (Invitrogen) respectively. 2mt8RP, Fura-Red and Indo-1 were used at single excitation and emission wavelengths. Either dye was dissolved in DMSO (4mM) containing 4% F127-Pluronic. Cells were loaded with Fura-Red by incubation with 4μM of the dye in the extracellular solution for 30 min. Imaging experiments were performed on an Olympus IX-71 microscope with UPlanFL N ×40 magnification objective. For acquisition, an F-View-II camera and MT-20 excitation system equipped with a Hg/Xe arc lamp were used, under control of CelÎR software (Olympus). Excitation/emission wavelengths were (nm): 410/535 (2mt8RP), 490/630 (Fura-Red), 490/535 (Perceval). Images were acquired at a frequency of 0.2 Hz with typical excitation times of 10 ms. The acquisition of the fluorescence and electrophysiological data was synchronized using TTL pulses. Imaging data was background-subtracted, analysed and presented as F/F_0_ (Perceval) and F_0_/F (Fura-Red, 2mt8RP). Whole cells were selected as regions of interest (ROI) to minimize the effect of the cell drift. For cell clusters, only the patched cell was included in the ROI. Every [Ca^2+^] recording was subjected to the dynamic range control by applying, at the end of the trace, solutions containing 10 μM ionomycin: “Ca^2+^-free” (0.5 mM EGTA), “Ca^2+^-max” (5 mM Ca^2+^). For the [ATP/ADP]_cyt_ recordings the dynamic range was controlled by high glucose (maximum after >30 min of exposure) and 2µM carbonyl cyanide 4-(trifluoromethoxy)phenylhydrazone (FCCP; minimum).

### Measurements of TMRE fluorescence

Cells were loaded with 7 nM TMRE for 60min at 3 mM glucose. Confocal imaging was performed in bath solution (see below) initially containing 3mM glucose, using a Zeiss microscope fitted with a Plan Apochromat x63 n. a. 1.4 oil immersion objective and equipped with Yokogawa CSU22 spinning disk module. The TMRE fluorescence signal was excited at 563 nm using a solid-state laser. Emission at 600 nm was registered using Hamamatsu ImagEM EM-CCD camera. The calculations of Ψ_m_ were done on the basis of the ratio of mitochondrial and cytosolic fluorescence, as was outlined in [48].

### Electrophysiology

Electrophysiological recordings and stimulation were done in whole-cell perforated-patch configuration, using an EPC9 patch-clamp amplifier controlled by Pulse acquisition software (HEKA Elektronik). The pipette tip was dipped into pipette solution, and then back-filled with the same solution containing 0.17 mg/ml amphotericin B. Series resistance and cell capacitance were compensated automatically by the acquisition software. Recordings, triggered by the TTL pulse, were started in current-clamp mode, and the depolarization of the plasma membrane was monitored simultaneously with [Ca^2+^] and [ATP/ADP]_cyt_, in response to a glucose step from 3 to 17 mM. To monitor the input resistance, the protocol included 10-ms injections of repolarising 10-pA current applied every 20s. The parameters of the current injections were chosen to minimise their effect on the glucose-induced electrical activity. To control V_m_ and impose electrical stimulations, the mode was periodically switched to voltage-clamp [49]. V_m_ was held at the value of −70 mV, with 0.5 Hz +5/−10 mV pulses to monitor the K_ATP_ conductance (see Suppl. Fig. S2B). The electrical stimulation was deemed to mimic the naturally occurring bursts of action potentials and comprised of 5-s depolarization trains to −30 mV containing 25 ramps of 100 ms to 0 mV and back (Suppl. Fig. S2B). Data were filtered at 1 kHz, and digitised at 2 kHz. G_m_ was normalized to cell capacitance to account for cell size.

### Experimental solutions

The pipette solution contained (mM): 76 K_2_SO_4_, 10 NaCl, 10 KCl, 1 MgCl_2_, 5 HEPES (pH7.35 with KOH). The extracellular bath solution, referred in text as “EC” contained (mM): 120 NaCl, 4.8 KCl, 24 NaHCO_3_ (saturated with CO_2_), 0.5 Na_2_HPO_4_, 5 HEPES (pH 7.4 with NaOH), 2.5 CaCl_2_, 1.2 MgCl_2_. All experiments were conducted at 32–33°C and the bath solution was perifused continuously.

### Data analysis

Imaging data was analysed using CelÎR (Olympus) and ImageJ (Wayne Rasband, NIMH). The simultaneous recordings were combined together and analysed using Igor Pro (Wavemetrics). The results are presented as mean±SEM. Mann-Whitney U-test and Wilcoxon's paired test were used to assess the statistical significance of the differences between the independent and dependent samples, respectively.

## Supporting Information

Figure S1 **Expression patterns of Perceval and 2mt8RP.** ***A***: A two-cell pancreatic β-cell cluster was infected with Perceval (48 h, λ_ex_ = 490 nm, λ_em_ = 535 nm) and incubated with Fura-Red (30 min, λ_ex_ = 490 nm, λ_em_ = 630 nm). ***B***: A three-cell pancreatic β-cell cluster was infected with 2mt8RP (48 h, λ_ex_ = 490 nm, λ_em_ = 535 nm) and loaded with Fura-Red (30 min, λ_ex_ = 490 nm, λ_em_ = 630 nm).
(TIF)

Figure S2 **Imaging ATP dynamics in single β-cells. Effects of pH, analysis of kinetics.** ***A***: Comparison of the effects of glucose and pH on the Perceval fluorescence. 17 mM glucose was applied to the cell, followed by 140 mM K^+^ plus 10 μM nigericin solutions of the indicated pH. ***B***: Schematic of the depolarisation protocol (single burst). ***C***: The first phase of glucose-induced [ATP/ADP]_cyt_ increase and the decrease in G_m_ were closely associated in time. G_m_ was calculated from I_m_ traces (Fig. 2B, inset). The pairs of signals (n = 12) were normalised by the range of change during the first phase of ATP elevation.
(TIF)
