# Supplementary figures and images for: The Mitochondrial Ca2+ Uniporter MCU Is Essential for Glucose-Induced ATP Increases in Pancreatic β-Cells
Source: PLoS One. 2012 Jul 19;7(7):e39722. doi: 10.1371/journal.pone.0039722 (PMC3400633; doi:10.1371/journal.pone.0039722)

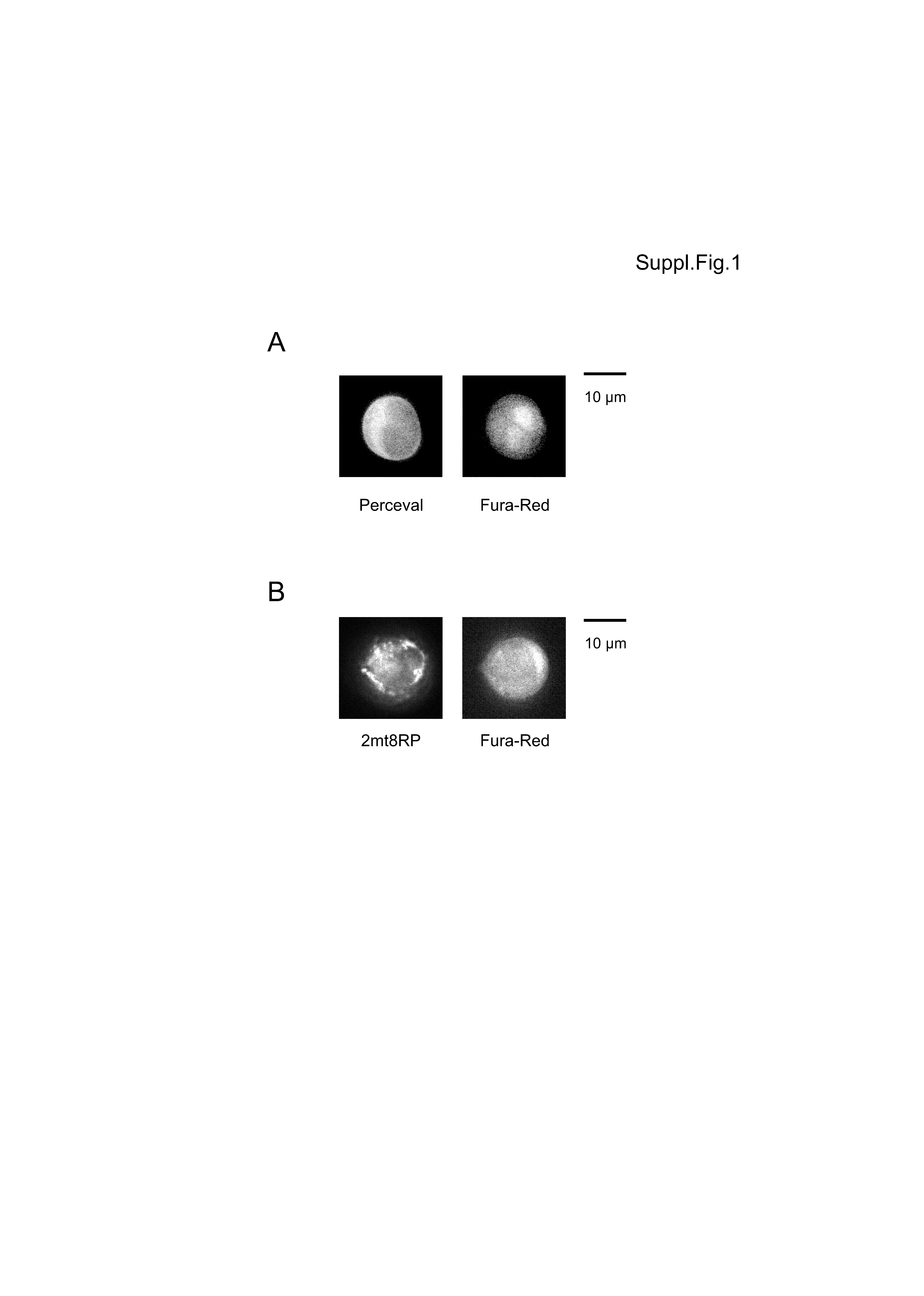

Supplement: Figure S1 — Expression patterns of Perceval and 2mt8RP. A: A two-cell pancreatic β-cell cluster was infected with Perceval (48 h, λex = 490 nm, λem = 535 nm) and incubated with Fura-Red (30 min, λex = 490 nm, λem = 630 nm). B: A three-cell pancreatic β-cell cluster was infected with 2mt8RP (48 h, λex = 490 nm, λem = 535 nm) and loaded with Fura-Red (30 min, λex = 490 nm, λem = 630 nm). (TIF) [file pone.0039722.s001.tif]

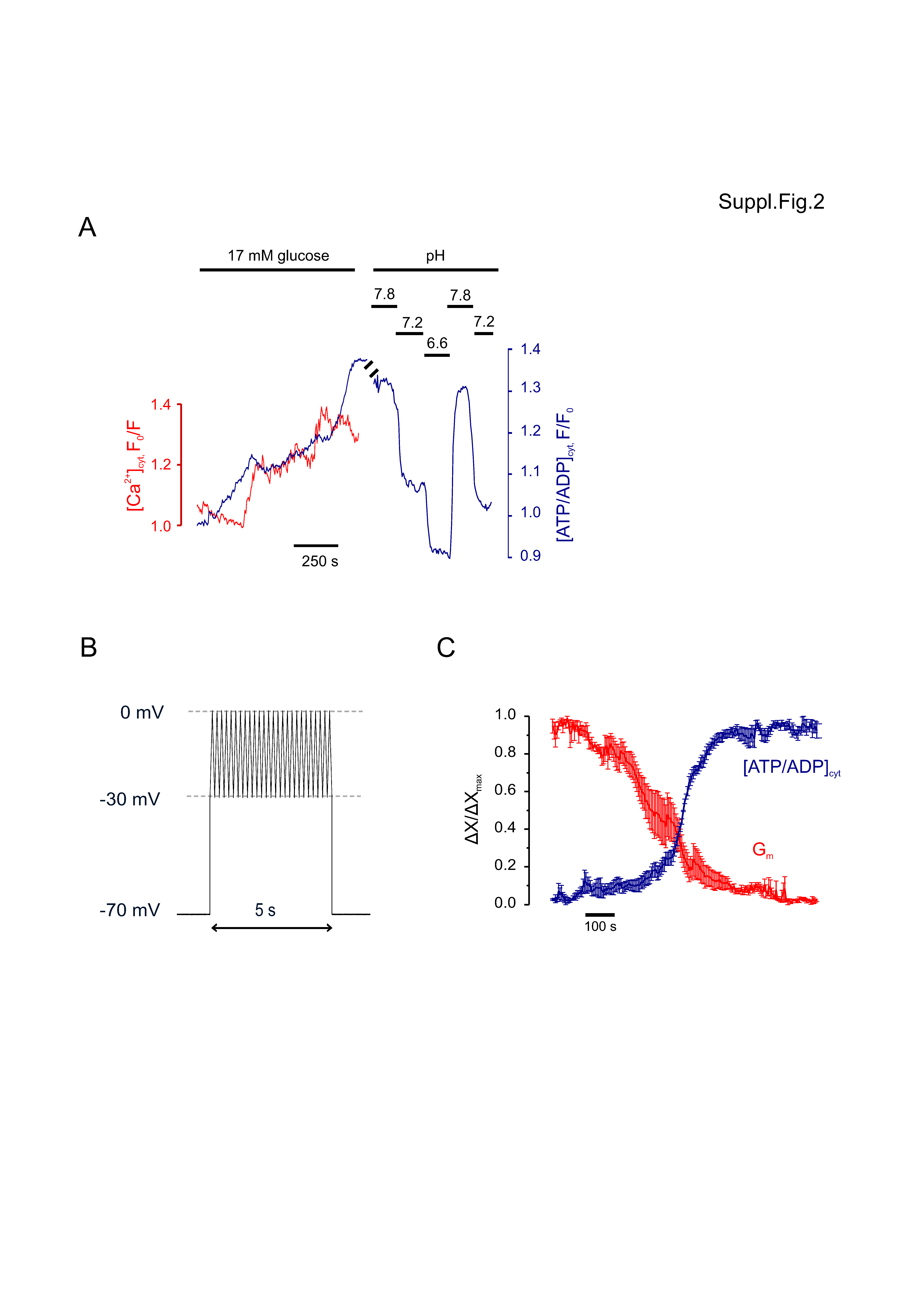

Supplement: Figure S2 — Imaging ATP dynamics in single β-cells. Effects of pH, analysis of kinetics. A: Comparison of the effects of glucose and pH on the Perceval fluorescence. 17 mM glucose was applied to the cell, followed by 140 mM K+ plus 10 μM nigericin solutions of the indicated pH. B: Schematic of the depolarisation protocol (single burst). C: The first phase of glucose-induced [ATP/ADP]cyt increase and the decrease in Gm were closely associated in time. Gm was calculated from Im traces (Fig. 2B, inset). The pairs of signals (n = 12) were normalised by the range of change during the first phase of ATP elevation. (TIF) [file pone.0039722.s002.tif]
